# Reconstructing the professional identity of rural pre-service teachers via an English pedagogy course: A case study

**DOI:** 10.1371/journal.pone.0358864

**Published:** 2026-09-22

**Authors:** Jun Chen, Xun Wan, Yu Zhou

**Affiliations:** 1 School of Foreign Languages and Literatures, Chongqing Normal University, Chongqing, China; 2 No.3 Middle School of Chengdu, Chengdu, China; 3 School of Foreign Languages and Literatures, Chongqing Normal University, Chongqing, China; UTM Skudai: Universiti Teknologi Malaysia, MALAYSIA

## Abstract

Teacher Professional Identity is critical for rural teacher recruitment and retention, but effective pathways for its construction remain limited. This study examines whether and how a holistically designed English pedagogy course, integrating theoretical learning and micro-teaching practices with guided reflective discourses, shapes the professional identities of rural pre-service teachers. A longitudinal study spanning one academic year was conducted with three rural pre-service teachers enrolled in the Excellent Teacher Program at a normal university in western China. Data were collected through semi-structured interviews and reflective journals. Drawing on Varghese et al.’s identity-in-practice and identity-in-discourse as a conceptual lens, and Wenger’s three modes of belonging as explanatory tools, our dual-layered framework reveals two key transformations. First, the course significantly transformed participants’ initially underdeveloped Teacher Professional Identity (self-doubt, narrow grammar and vocabulary focus) into a broader, more confident and committed identity. Two patterns of change were identified: broadening of pedagogical vision from discrete skills to holistic design, and affective repositioning from fear and doubt to confidence and clarity. Second, the course functioned as a discourse-rich community of practice wherein three mechanisms operated to facilitate identity (re)formation: engagement through micro-teaching and peer collaboration, imagination through exposure to contemporary theories, and alignment through guided reflections linking practice to professional standards. The study demonstrates that a campus-based, holistically designed pedagogy course can serve as an effective pathway for the professional identity construction of rural pre-service teachers, particularly in contexts where quality field placements are not feasible. It also highlights the need to integrate such on-campus training with authentic rural experiences (e.g., short-term placements) to translate identity gains into sustained commitment to rural teaching. These findings offer insights for teacher education policy and practice, with implications for addressing global challenges in rural teacher retention.

## Introduction

Teacher Professional Identity (TPI) is widely recognized as a cornerstone of teachers’ motivation, job satisfaction, and long-term commitment to the profession [1–5]. Low TPI has been directly linked to teacher shortages and high turnover rates in rural schools [6–9], where attracting and retaining qualified teachers remains a global concern [10]. Consequently, enhancing TPI among rural pre-service teachers (PSTs) is widely seen as a key strategy for addressing staffing deficits, improving educational quality, and promoting equity in these areas [7,11,12].

Given the importance of TPI for rural PSTs, existing studies have highlighted teaching practicum as the primary site of PSTs’ identity construction [13–16]. TPI formation is regarded as the central outcome of “learning to teach” process [17–22]. During this process, PSTs’ TPI can be reified through their reflective practice in a community of practice [23, 24], where positive support from and interactions among teacher educators, mentors and peers can enhance their teacher agency for critical reflection, especially through critical incidents [25, 26]. However, such TPI formation heavily relies on high-quality field placements, which are often difficult to secure in under-resourced rural areas worldwide. For many rural PSTs in western China, in particular, long-term, well-supervised practicums in local rural schools are rarely available, constrained by limited educational resources and a general shortage of qualified in-service mentors [10]. Against this background, university-based teacher training courses serve as an alternative pathway for rural PSTs’ identity construction. However, the role of such courses in this regard remains under-explored [27]. Furthermore, few studies have investigated effective strategies for preparing teachers specifically tailored for rural teaching assignments [6,11,28], and productive pathways for shaping PSTs’ TPI are still limited [29].

To address these gaps, this study presents a longitudinal case study of three rural pre-service English teachers in a Chinese university, tracking their TPI reconstruction over a full academic year in a holistically designed English pedagogy course. We aim to unpack how this on-campus pedagogy course, which integrates theoretical learning, micro-teaching practice and guided reflection, can facilitate rural PSTs’ TPI formation when high-quality field placements are not available. Understanding how the English pedagogy course influences TPI construction may improve teacher training programs [30] and support the recruitment and retention of English teachers in rural schools. This paper thus contributes to the discourse on pre-service teacher education and identity development in rural China and similar regions globally. In doing so, it responds to the call for research that moves beyond practicum-only frameworks and explores curriculum-embedded strategies for shaping resilient professional identities among future rural teachers.

## Literature review

### Previous studies on pre-service teacher professional identity

Much of the research has focused on how PSTs’ identities evolve during their teacher preparation programs, with the teaching practicum receiving the most sustained empirical examination [13–16]. Research within the practicum context consistently highlights the pivotal role of mentoring and institutional environments in (re)shaping PSTs’ professional identity. Supportive mentoring and the fulfilment of practicum expectations have been found to enhance pre-service teachers’ self-confidence and sense of professional self [17,26,31], whereas restrictive mentoring practices and rigid institutional norms can elicit negative emotions and constrain identity development [32–34]. These findings suggest that the practicum’s influence on identity is highly context-dependent and not inherently positive. However, the mentoring resources and institutional support that appear crucial for positive identity growth are precisely what tend to be lacking in under-resourced rural schools, the very context for which this study seeks to prepare pre-service language teachers. Furthermore, the practicum-centric orientation has inadvertently narrowed the understanding of how other components of teacher education, particularly structured, curriculum-based interventions, might contribute to identity construction.

In contrast, studies on curriculum-based approaches have demonstrated that intentional course design can foster identity development, with teacher educators and pedagogy curricula more specifically recognized as pivotal factors shaping PSTs’ professional identities [35–38]. The pedagogy curriculum, as a mandatory element of teacher education programs, is theorized to contribute to TPI formation by providing structured opportunities for dialogue and critical reflection on professional matters [39], as well as by engaging PSTs with didactic and pedagogical learning experiences central to their emerging sense of professional self [40]. For example, Tsybulsky and Muchnik-Rozanov identified a positive relationship between PSTs’ participation in an online project-based learning course, designed as a pedagogy of practice, and their professional identity development [41].

However, despite their respective contributions, these two strands of research have rarely been brought into productive dialogue. The practicum literature has illuminated the context in which identity is shaped, but offers limited guidance on how teacher education programs can systematically prepare PSTs to navigate those contexts before immersion. Conversely, curriculum-based studies have demonstrated the potential of structured pedagogy for identity formation [42], yet their integration into subject-specific courses, particularly those preparing language teachers for the distinctive challenges of rural schools, remains largely unexplored. What remains unclear from both lines of inquiry, however, is not merely whether curriculum-based interventions can foster identity, but how structured practice and guided reflection actually work to construct identity, a question that requires theoretical elaboration. This disconnection is particularly consequential for rural teacher education, where the supports that sustain positive identity growth during the practicum are often absent, and where pedagogy courses may thus represent a uniquely positioned, and under-researched pathway for proactive identity construction [28]. Such structured curricula, when combined with guided reflection, provide a safe space for PSTs to experiment with professional identities and develop coping strategies, an outcome that practicum-only models cannot guarantee.

Theoretically, the facilitative role of structured practice and guided reflection can be understood through a socio-cultural lens. Structured practice, such as micro-teaching or simulated teaching tasks, provides PSTs with repeated opportunities to enact teaching roles in a friendly environment, thereby reducing anxiety and building self-efficacy [43]. Guided reflection, particularly when scaffolded by teacher educators and peers, operates as a mediational tool that enables PSTs to externalize their tacit beliefs, confront dissonance, and reconstruct their professional self-understanding. This dialogic process, embedded in a community of practice, facilitates the internalization of professional norms and values [24], turning isolated experiences into cooperative professional growth. Critical incident reflection, for example, has been advanced as a structured dialogic process embedded within TESOL teacher education coursework, showing that this multi-layered reflective engagement cultivates professional awareness and facilitates identity negotiation by inducting PSTs into a“dialogic activity within the self” [26,29]. The power of this approach lies in its systematic design: it transforms reflection from a solitary and often superficial exercise into a scaffolded, intersubjective process, one in which teacher educators’ guidance can direct PSTs’ attention to critical incidents, provide alternative interpretations, and thereby expand their interpretive repertoires and enhance their agency to negotiate identity conflicts [25]. In this sense, structured practice and guided reflection are not merely supplementary activities, they are constitutive mechanisms that enable PSTs to develop a resilient and adaptive professional identity before they encounter the unpredictable realities of rural classrooms.

If PSTs are to develop a resilient professional identity suitable for rural schools, teacher education programs cannot rely solely on the practicum, they must also leverage structured curricula to equip PSTs with the reflective and agentive capacities to negotiate predictable structural challenges before they enter the field. The present study seeks to bridge the intersecting gap between practicum-focused and curriculum-focused inquiry by examining how an English pedagogy course with a more holistic design shapes the TPI development of rural pre-service English teachers.

### A framework for understanding teacher professional identity

Teacher Professional Identity (TPI) refers to how teachers perceive themselves as professionals, developing through an ongoing, dynamic process of professional growth [35]. TPI is continuously redefined through negotiation, shaped by both internal and external influences [35,36,44–48]. Rather than attempting to construct a new, overarching model of TPI, this study adopts a dual-layered analytical framework. At the first layer, we draw on Varghese et al.’s identity-in-practice and identity-in-discourse [49] as a conceptual lens to trace transformations in participants’ professional selves. At the second layer, we adopt Wenger’s three modes of belonging—engagement, imagination, and alignment [24]—as explanatory tools to uncover the mechanisms through which the English pedagogy course facilitates these changes. Together, these two layers offer a productive framework for examining how the activities and dialogues embedded in a course may serve as a site for TPI construction among rural pre-service teachers.

The concept of identity-in-practice views identity formation as an action-oriented process that occurs through participation in the concrete tasks and shared activities of a community [24,49]. From this perspective, “identification takes place in the doing” [24, p. 193]. Varghese et al. illustrated this through a study of bilingual pre-service teachers whose professional identities were formed not merely through coursework, but also through their evolving modes of participation in the professional community [49]. She found that these teachers enacted their identities along a continuum, from being classroom-focused practitioners, to becoming advocates who extended their roles beyond the school, demonstrating that identity formation is fundamentally linked to engagement in practice and access to professional communities [38,49]. Wenger theorizes this process through three modes of belonging: engagement (collaborative problem-solving with peers and mentors), imagination (forming a self-image that extends beyond immediate experience), and alignment (connecting local practices with broader professional goals) [24,50], which will be elaborated later as explanatory mechanisms for how the course facilitates TPI change.

The concept of identity-in-discourse, in contrast, foregrounds the role of language in constructing, maintaining, and negotiating teacher identities [49]. This perspective posits that identity is not merely reflected in talk but is actively constituted through it. Trent operationalized this in a study of early-career English teachers in Hong Kong [51], using Fairclough’s tools of modality and evaluation [52] to show how teachers discursively positioned themselves. For example, one teacher’s declaration that “as teachers, we must be well prepared for every class” [51, p. 8] revealed a strong commitment to a specific professional self, while also exposing a conflict with the competing discourse of “time constraints” imposed by institutional demands. This analysis demonstrates how teachers’ professional identities are discursively constructed through the commitments and evaluations they express in language, and how these identities are sites of struggle between a teacher’s preferred sense of self and the institutional discourses that position them otherwise [38,51]. For pre-service teachers, the structured dialogues, reflective writing, and peer discussions that occur within a pedagogy course are thus not merely vehicles for expressing identity, but are the very discursive mechanisms through which identity is negotiated and reconstructed [26,29].

These two dimensions are not mutually exclusive but deeply intertwined, identities enacted in practice are articulated, made sense of, and negotiated through discourse [19,49]. This conceptual lens is therefore particularly relevant to the present study as the English pedagogy course under investigation is designed holistically, integrating theoretical learning, micro-teaching practice and reflective dialogue. The identity-in-practice dimension enables us to trace changes manifested through observable behavioral participation, such as pre-service teachers’ evolving engagement in micro-teaching tasks, collaborative planning, and problem-solving activities within the course community. Concurrently, the identity-in-discourse dimension allows us to capture changes as they are articulated, negotiated, and reconstructed through language, evidenced in reflective journals and dialogues. While the dual lenses of identity-in-practice and identity-in-discourse allow us to observe what changes in participants’ professional selves, they do not, by themselves, explain how such changes are generated.

To uncover the mechanisms behind these changes, we turn to Wenger’s community of practice framework, which specifies three modes of belonging [24]—engagement, imagination, and alignment—through which identity is formed and reformed within a learning community. Specifically, engagement helps us examine how collaborative micro-teaching and peer feedback sessions foster active participation and mutual problem-solving; imagination enables us to analyze how reflective dialogues allow rural PSTs to construct possible future selves beyond their immediate teaching contexts; and alignment allows us to assess how course discourses connect PSTs’ local practices to broader professional standards and institutional expectations.This ensures our analysis identifies course outcomes and illuminates the underlying explanatory mechanisms.

This dual-layered framework directly informs our research questions. RQ1 focuses on identifying what changes occurred in participants’ TPI after the course, while RQ2 examines how the course produced these changes. The specific research questions are as follows:

RQ1: What changes may occur in the Teacher Professional Identity (TPI) after completing the English pedagogy course?
RQ2: How did the English pedagogy course impact the development of PSTs’ TPI?

### The study

#### Context.

Educational reforms worldwide have focused on attracting and retaining teachers, particularly in rural areas, where highly qualified teachers are in short supply [38]. Meeting the needs of rural learners, who face disadvantages due to poverty and isolation, requires specialized training [28]. In response, China has implemented a range of initiatives to prepare and support qualified teachers for rural areas. One such initiative is the Excellent Teacher Program, which is carried out by provincial normal universities tasked with preparing teachers for formerly impoverished and remote areas in central and western China. This study is conducted at one such university in western China, focusing on pre-service English teachers enrolled in this program.

Within this program, the English pedagogy course, a compulsory core course specifically designed to prepare PSTs for rural English teaching, serves as a key intervention. Spanning one academic year (from the second semester of the sophomore year to the first semester of their junior year), the course integrates theoretical instruction with practical training through weekly lecture-based class and one micro-teaching session. Theoretical instruction covers theories on curriculum standards, textbook analysis, lesson implementation, and assessment, while practical sessions focus on lesson design and classroom application.

PSTs in the Excellent Teacher Program complete a four-year undergraduate program and are required to teach in designated rural areas for at least six years after graduation. But the tension lies in that these PSTs, having had minimal exposure to rural school realities prior to the program and encountering constrained practicum conditions with limited access to experienced mentors and quality resources, often find their rural English teacher identity largely underdeveloped before they formally enter the profession.

In this context, through shared practice and reflective discourses with peers guided by teacher educators, the English pedagogy course provides pre-service rural English teachers with structured opportunities to construct and negotiate their TPI, thus serving as a key site where TPI development is facilitated through the interaction between pedagogical knowledge acquisition and reflective teaching practice.

#### A case study.

This study employed a single embedded case study design [53] with an instrumental purpose [54] to investigate how an English pedagogy course contributed to the development of pre-service language teachers’ professional identities. The case was the English pedagogy course within the Excellent Teacher Program, bounded by a specific institutional context and a one-year period. The three PSTs from urban, suburban, and rural backgrounds served as embedded units of analysis, allowing the study to examine variation within the single case.

A single embedded case study was particularly appropriate for this research, as it facilitated an in-depth understanding of how individual experiences within the English pedagogy course contributed to the construction of TPI over time. Furthermore, the study’s context aligned with the concept of a bounded case, defined by the specific time and setting in which the participants’ experiences were situated.

#### Participants.

To ensure representativeness and relevance, this study used convenience access to the research site and purposive sampling for participant selection.

First, convenience access was used to gain entry to the research site through teacher educators of the English pedagogy course who were known to the researchers. From the accessible pool of prospective PSTs, purposive sampling was then used to select three participants who could provide rich and diverse insights into the phenomenon [55]. The three participants were selected to represent urban, suburban, and rural backgrounds, respectively, and served as embedded units of analysis.

The participants were around 20 years old, they were classmates and had committed to teaching in rural areas for six years after graduation. They represented urban, suburban, and remote regions, reflecting the diversity of recruiting prospective English teachers for rural areas. All participants specialize in English Language Teaching and began the English pedagogy course in their sophomore year, and had not received any prior teacher education (see Table 1).

**Table 1:** Demographic characteristics of three prospective English teachers in rural areas.

Participant	Major	Grade	Gender	Hometown	Site of the assigned rural school
Ada*	English Teaching	Sophomore	F	Urban	Remote rural area
Betty*	English Teaching	Sophomore	F	Suburban	Remote rural area
Cooper*	English Teaching	Sophomore	M	Remote rural area	Remote rural area

*. pseudonym names.

#### Data collection.

The study was conducted from September 2024 to January 2025. Data for this study were collected from two sources: (a) pre- and post-course semi-structured interviews and (b) reflective journals completed during and after the course.

**Semi-structured interviews**. To explore how PSTs construct their identity, each participant took part in two group interviews conducted with all three participants together: one before and one after the English pedagogy course. The interviews, conducted in Chinese, lasted approximately 60 minutes each. In designing the interview questions, we drew on Trent’s integrated framework for investigating language teacher identity construction [51] as a guiding analytical and conceptual basis. The first author asked participants to reflect on their identity as prospective rural English teachers and the impact of the pedagogy course on their TPI. Follow-up questions were asked to clarify responses. The second and third authors recorded the interviews, remaining mostly silent to avoid interrupting. With participants’ consent, all interviews were audio-recorded and transcribed verbatim in Chinese. The first author then translated the relevant data into English. To ensure translation accuracy, the English translations were checked against the original Chinese transcripts by the research team, and discrepancies were discussed and resolved through consensus. Unnecessary data were removed during transcription to refine the textual data.

Each interview targeted specific research questions (see Table 2). The first interview focused on the three participants’ initial cognition of becoming rural English teachers, aiming to understand their perceptions of the profession prior to taking the English pedagogy course. Conducted after the one-year course, the second interview explored changes in participants’ perceptions of being rural English teachers and possible shifts from their initial views. It also examined how the English pedagogy course shaped their perceptions and contributed to the development of their professional identities.

**Table 2:** Data collection overview.

Instrument	Purpose	Time Point	Focus
First Interview	For RQ 1	Pre-course	Initial perceptions of being a rural English teacher
Second Interview	For RQ 1 and RQ 2	Post-course (after one year of pedagogy learning)	Re-cognition of the profession English pedagogy course’s impact on identity development
Reflective Journals	For RQ 1 and RQ 2	Throughout and immediately after the course	Ongoing identity reflections

**Reflective journals**. To explore PSTs’ identity development during and after the course, they were required to maintain reflective journals written in Chinese. The journals were guided by open-ended prompts designed to encourage reflection on learning experiences, identity-related changes, and pedagogical understandings throughout the English pedagogy course. These reflective entries served as a supplementary data source to complement the interview data.

The semi-structured interview protocol and reflective journal guidelines were developed based on a review of existing literature on TPI and rural teacher education. To enhance content validity, the instruments were reviewed and refined through consultation with experienced teacher educators in English pedagogy. A pilot interview was conducted with a non-participant PST to test clarity, relevance, and flow of questions, leading to further refinement of wording and sequencing. Additionally, data triangulation between interviews and reflective journals was employed to strengthen the credibility and trustworthiness of the findings.

#### Data analysis and trustworthiness.

To address the research questions, hybrid inductive-deductive thematic analysis was conducted on the interview transcripts and reflective journals (see Table 3), following Braun and Clarke’s six-phase approach: familiarization with the data, generating initial codes, searching for themes, reviewing themes, defining and naming themes, and producing the report [56].

**Table 3:** Thematic analysis examples.

Initial code	First-order theme	Second-order theme
**RQ1: What changes may occur in the Teacher Professional Identity (TPI) after completing the English pedagogy course?**		
Teaching grammar and vocabulary (interview transcripts) Minimal need for pedagogy knowledge (interview transcripts) Following the previous English teachers at their secondary schools (interview transcripts) High demands of ELT (interview transcripts) Worries about being qualified English teachers (interview transcripts, reflective journals)	Simplistic view of ELT Professional self-doubt	Underdeveloped TPI
**RQ2: How did the English pedagogy course impact the development of PSTs’ TPI?**		
Learning advanced teaching theories (interview transcripts, reflective journals) Integrating theories into teaching practices (interview transcripts, reflective journals) Teacher educator’s guidance and inspiration (interview transcripts, reflective journals) Discussions and reflections with peers (reflective journals)	Deepen understanding of ELT	The Influence of course on TPI

The analysis combined inductive coding from the data with deductive interpretation informed by the conceptual framework. Initially, codes and sub-themes were generated inductively by identifying recurring patterns across the interview and journal data. For example, codes such as “teaching grammar and vocabulary,” “reliance on English skills,” and “minimal need for pedagogical knowledge” were grouped into the sub-theme “simplistic cognition of TPI,” while codes related to “pedagogical knowledge,” “teaching theories,” and “teaching confidence” were grouped into “more professional views of TPI.” These data-driven categories were then compared and refined to identify broader themes concerning changes in TPI. The subsequent excerpt from Ada’s initial interview exemplifies the coding process:

I believe that the work of an English teacher might be relatively simpler, focusing more on aspects such as vocabulary and grammar. (Ada, Interview 1).

In this statement, Ada articulated her straightforward perception of being an English teacher, which was shaped by her previous learning experiences. This response was categorized as “teaching vocabulary and grammar”, falling within the sub-theme of “Simplistic View of ELT.”

For this first research question (RQ1)—what changes occurred in TPI—these emergent themes were further examined through the deductive lenses of identity-in-practice and identity-in-discourse [49] to trace transformations in participants’ enacted and articulated professional selves. For the second research question (RQ2)—how these changes were produced—the deductive analysis was guided by Wenger’s three modes of belonging (engagement, imagination, and alignment) [24] as explanatory tools. These concepts helped interpret the generative mechanisms embedded in the course design, including how micro-teaching practice, peer collaboration, theoretical input, and guided reflective discourse contributed to participants’ identity reconstruction. Thus, while the specific codes and patterns emerged from the data, their interpretation was systematically informed by the differentiated conceptual framework.

To ensure trustworthiness, this study employed the criteria proposed by Lincoln and Guba, including credibility, transferability, dependability, and confirmability [57]. To establish credibility, data were collected from two different sources and triangulated during the analysis to ensure that the findings were appropriately categorized into relevant and coherent themes. Transferability was addressed through the use of thick description, providing detailed accounts of the research context, participants, and the English pedagogy course, allowing readers to determine the applicability of the findings to similar contexts. Dependability was established through prolonged engagement with the participants and data, as well as participant checking and peer debriefing [58]. The three researchers independently coded the data, then reached a consensus through discussion, revisiting the raw interview and journal data. They also conducted multiple discussions with participants to verify details. Confirmability was established by grounding the findings in the data. During the coding process, the researchers were influenced by the findings of Trent [51] while maintaining an open stance toward the data, allowing patterns and themes to emerge inductively rather than being predetermined. Ultimately, through a case-study analysis of identity changes, the study captured authentic transformations in the TPI of pre-service rural English teachers and how the English pedagogy course influenced these changes.

### Ethics statement

This study was approved by the Institutional Review Board of Chongqing Normal University (Approval No. AREC2023SFLL031606). Written informed consent was obtained from all participants.They were informed that participation was voluntary, anonymous, and that their responses would be used for research publication and securely stored to ensure confidentiality.

## Results

### Changes in TPI after the English pedagogy course

The three participants’ narratives reveal a shared trajectory of TPI transformation, from simplistic, fragmented, or fear-laden conceptions towards more professional, confident, and holistic understandings of being an English teacher. These changes are traced through the dual lenses of identity-in-practice and identity-in-discourse [49]. Across the three cases, two overarching patterns emerge: broadening of pedagogical vision and affective repositioning towards professional competence.

#### Broadening pedagogical vision: From discrete skills to holistic design.

The three participants’ initial perception of English teaching was marked by the belief that it was comparatively straightforward, concentrating mainly on discrete linguistic components such as vocabulary and grammar, a view reflecting what Richards terms a limited, skill-based conception of language teaching [22]. For them, teaching English required little pedagogical knowledge, focusing mainly on basic language skills. In their initial interviews, Ada perceived English teaching as “simple” as transmitting “vocabulary and grammar,” Betty “didn’t have a very clear understanding of how English teachers teach,” and Cooper just simplified the goal of teaching as “to teach students how to do well in exams.” This initial stance suggests that for them, as for many PSTs with minimal exposure to rural teaching realities, language proficiency alone was mistaken as the primary qualification for teaching, with little recognition of pedagogical content knowledge.

However, these perspectives transformed markedly after the course, becoming more refined and congruent with the intricate realities of teaching. Ada now articulated that “there’s still a lot for an English teacher to learn,” referencing curriculum standards, textbook analysis, and student needs as integral to lesson planning. Betty stated that the contemporary English teaching theories introduced by the course transformed her professional self-perception. Cooper gained a more comprehensive understanding of teaching as a professional and purposeful practice. The following words from Cooper’s second interview exemplify the shared development across the three participants:

Now, it’s more planned. We design the whole lesson with relevant components in mind. The way we think about teaching has matured. I don’t approach problems in such a naive way anymore, I think about them more thoughtfully and thoroughly. (Cooper, Interview 2)

The shift from “simple” to “complex” in their narratives signifies that they have obtained a deeper understanding towards language teaching, which needs more holistic and deliberate design. It represents a reconfigured identity-in-practice, wherein their evolving participation in lesson design and micro-teaching positions them as designers of learning rather than transmitters of knowledge. Notably, their frequent use of “we” (“we need to study... we need to develop”) suggests an emerging alignment with the professional community’s collective standards as illustrated by Wenger (1998), moving beyond isolated, individualistic notions of teaching.

#### Affective repositioning: from fear and doubt to confidence and clarity.

Besides an oversimplified view of language teaching, their initial identities were also characterized by anxiety and a sense of inadequacy. Their first interviews revealed a discourse of fear: “I think it is quite difficult,” “I am pretty scared,” and “I am a bit afraid.” For Betty, it is coupled with a belief that English teaching demands extroversion, a trait she felt she lacked as evidenced in her statement: “English seems to require someone outgoing, like an extroverted person. Our previous English teacher is good at speaking, so I am a bit afraid.” This anxiety and fear stemmed partly from their limited knowledge of teaching and partly from their preconceptions about what a teacher should be like. Their understanding of being an English teacher prior to the course was largely derived from their English learning experiences at high school, which they modeled on their own English teachers. This can be exemplified in Cooper’s typical statement: “I don’t have much of an opinion. It might be similar to how my middle and high school teachers taught.”

Nevertheless, exposure to novel teaching concepts and methodologies during the course enabled them to gain clarity and foster confidence in being an English teacher. The course functioned as a discursive space for identity renegotiation. Ada developed a greater sense of agency in her pedagogical decisions as evidenced in her second interview: “We’re learning much more than that now...we need to...develop teaching plans based on the different needs of the students as a whole.” Her tone reflects a growing, well-founded confidence, built through systematic learning and practical experience. Betty’s post-course reflection, “teacher educators have instilled the latest teaching concepts... this has had a big impact on me,” signals not only cognitive change but affective repositioning. Her fear is replaced by gratitude and clarity. Critically, she no longer frames teaching competence as innate personality (extroversion) but as learnable professional knowledge. Cooper viewed his previous passive exam-oriented belief of language teaching as “naive” and described his present approach of design as “mature,” “more thoughtful and thorough.” This cognitive turn indicates a reconstructed identity-in-practice: Cooper now sees himself as an agent of deliberate pedagogical decision-making, no longer a passive imitator. His use of “we design the whole lesson with relevant components in mind” suggests emerging alignment with the profession’s standards, mirroring Ada’s trajectory. Their transformation, in retrospect, resonates with what Wenger terms the imagination mode [24]—they began to envision a possible future self as a competent teacher, decoupled from their initial fear and doubt, and anchored instead in the systematic knowledge base provided by the course.

Furthermore, all three participants’ post-course discourse features a notable shift in pronoun use, from “I” and “my” to “we” and “us,” suggesting an emerging sense of belonging to a professional community, a key indicator of identity-in-practice aligned with Wenger’s notion of engagement, which will be taken up as an explanatory mechanism in the next section.

#### Mechanisms of TPI development: Engagement, imagination, and alignment.

Building on the observed changes, this section adopts Wenger’s (1998) three modes of belonging: engagement, imagination and alignment, as explanatory tools to uncover the mechanisms underlying the participants’ TPI reconstruction.

#### Engagement through structured practice and peer collaboration.

The weekly integration of theoretical instruction with micro-teaching sessions created what Wenger terms a community of practice [24], wherein participants engaged in collaborative problem-solving. Ada’s reflection “designing a whole lesson on your own...and then actually trying to teach it in front of the class has really helped improve my overall thinking.” exemplifies identity formation through engagement: professional self is enacted in the doing. Similarly, Betty noted that class discussions on teaching methods “provided context for understanding real teaching situations,” while Cooper described the practical component as “an important way for me to transform theoretical knowledge into actual teaching ability.”

Crucially, these accounts indicate that engagement operated not as isolated practice but as situated participation, micro-teaching was performed and critiqued within a peer community. This collective dimension is central to identity-in-practice: identification is not merely individual skill-building but a process of becoming a certain kind of practitioner recognized by and accountable to one’s community. The participants’ repeated references to peer observation and feedback suggest that the course functioned as a legitimate peripheral participation structure, where PSTs gradually moved from novices to more competent members of the teaching community.

#### Imagination through exposure to contemporary theories and possible selves.

The course’s theoretical content, particularly concepts such as “students’ core competence” and “alignment of teaching, learning and assessment” provided more than knowledge; it enabled participants to imagine alternative professional selves. Wenger’s imagination mode refers to forming a self-image that extends beyond immediate experience [24]. Ada’s reflective journal entry, “Advanced teaching concepts have been integrated throughout my learning process. I feel fortunate to have been exposed to current, progressive teaching ideas.” reveals that her identity became anchored not in past school experiences but in an aspirational professional future. Cooper’s metaphor of theory “opening a new door” similarly signals imagination at work: he now views English teaching as “a more scientific and systematic practice,” a formulation that distances him from his previous “naive” self and projects a future identity aligned with professional standards. Betty’s newfound clarity about the profession’s demands, explicitly referencing “core competence” in her reflections, further demonstrates that imagination operates through the discursive resources the course provides. In all three cases, the course offered not just tools for teaching but a vocabulary for self-definition, enabling PSTs to articulate who they aspire to become.

#### Alignment with professional standards and institutional discourses.

Alignment, connecting local practices to broader professional goals, emerges most clearly in participants’ post-course discourse. Ada’s reference to curriculum standards, textbook analysis, and student needs reflects her internalization of the course’s explicit professional frameworks. Cooper’s shift from “teaching how to do well in exams” to “designing the whole lesson with relevant components” signals a realignment of his practice from exam-oriented local norms to curriculum-standard-based professional expectations.

This alignment is not merely cognitive but discursive. Through the course’s reflective dialogues and written journals, participants learned to speak the language of the profession. Varghese et al. posit that identity is constituted through the commitments and evaluations expressed in language [49]. Ada’s declaration that a teacher must “study curriculum standards” “analyze textbooks and students’ needs” and Cooper’s assertion that teaching requires “thorough” thinking are not merely descriptions but performative acts of identity. Through such statements, they position themselves as professionals who subscribe to the community’s norms.

Crucially, these discursive performances functioned as active sites of identity negotiation. When Betty stated, “the teacher educator has instilled the latest teaching concepts in us,” she was not merely reporting a fact but discursively positioning herself as a receptive, transformed learner. Similarly, when Cooper wrote that his previous thinking was “naive,” he performed a break with his past identity, a discursive act of boundary-marking that solidified his new professional self. The teacher educator, as repeatedly acknowledged by participants, served as the primary agent of this discursive alignment, modelling professional language and providing evaluative feedback that reinforced the standards of the community. Ada’s admiration of her teacher educator as “knowledgeable, outgoing, and elegant”—an aspirational figure—further illustrates how the educator’s personal example provided participants with a concrete discourse model. This aligns with Karim et al., who argue that for PSTs, structured dialogues are the very mechanisms through which identity is negotiated and reconstructed [26,29].

## Discussion

This study aims to examine whether and how a holistically designed English pedagogy course could reshape the professional identity of rural PSTs enrolled in China’s Excellent Teacher Program. Drawing on the dual lenses of identity-in-practice and identity-in-discourse [49], and Wenger’s community of practice [24], our findings offer both encouraging evidence of identity transformation and a nuanced understanding of the mechanisms driving that transformation.

Prior to the course, the participants’ professional self-understandings were narrowly defined: Ada reduced teaching to vocabulary and grammar, Cooper saw it as exam preparation, and Betty doubted her suitability due to perceived personality mismatches. Their initial identities reflected a limited, skill-based conception of teaching [22]. After the course, however, these views gave way to a more holistic and deliberate understanding of teaching, accompanied by greater confidence and a strengthened sense of professional agency. This transformation was reconstructive rather than merely additive, encompassing cognitive, affective, and agentive shifts. These findings are consistent with prior work linking identity change to enhanced performance and commitment [26,35,36].

Our analysis reveals three intertwined mechanisms that explain how these changes were produced. Engagement was fostered through weekly micro-teaching sessions and peer collaboration, where identity was enacted “in the doing” [24, p. 193]. Unlike studies that locate identity construction primarily in field placements [17,31–34,59], our course demonstrates that simulated yet pedagogically authentic teaching tasks within a university setting can also effectively cultivate practice-based identity, a crucial advantage for rural PSTs with limited access to quality practicum schools. Imagination was activated through exposure to contemporary theories such as “students’ core competence” and “alignment of teaching, learning and assessment,” enabling participants to construct possible future selves beyond their past school experiences. This extends Trent’s observation that teacher identities are discursively built through expressed commitments, by demonstrating that imagination can be deliberately fostered within a course rather than left to chance [51]. Alignment emerged as participants internalized professional standards, referencing curriculum frameworks, textbook analysis, and student needs, and learned to speak the language of the profession through reflective dialogues. The teacher educator served as the primary agent of this discursive alignment, modelling professional discourse and providing evaluative feedback that reinforced community norms. This finding is consistent with Karim et al.’s argument that structured dialogues are the very mechanisms through which identity is negotiated and reconstructed [26,29].

Our findings make two contributions to the literature. First, whereas existing research often treats practicum-based and curriculum-based identity development as separate pathways [35,38], this study shows that a well-structured pedagogy course with a holistic design can constitute a productive pathway for TPI construction. By systematically integrating theoretical learning, micro-teaching practice, and ongoing reflective discourse within a university-based community of practice, the course offered a model where identity is forged through intertwined processes of practice and discourse rather than as separate tracks. Second, our findings challenge the view that pre-service teacher education has minimal impact on teaching approaches and self-perception [36]. This difference is likely attributable to the structural features of the Chinese teacher preparation program, a four-year undergraduate degree in which the English pedagogy course alone spans a full academic year. In contrast, many other countries offer one-year postgraduate programs, with only a few weeks of campus-based theoretical study and most training occurring in schools. This approach frames teacher preparation primarily as workplace training, often at the expense of sustained engagement with pedagogical theory [31,60]. Such a separation risks creating a divide between theoretical knowledge and practical know-how, a gap that can best be bridged through closer collaboration between universities and schools [60]. The campus-based course design in this study integrates theoretical instruction with regular micro-teaching, providing systematic and extended integration of theory and practice, a model of particular value for rural PSTs with limited field placement options.

Despite these positive gains, the course did not fully develop rural-specific preparedness. Two related limitations are worth noting in this regard. First, the course content focused on general English language teaching competence, and generic curriculum design--curriculum standards, textbook analysis, and lesson planning--rather than addressing the specific challenges of rural schools, such as multigrade classrooms, limited resources, or socio-economic deprivation. While participants developed professional confidence and pedagogical knowledge, they did not articulate how these would be adapted to rural realities. Second, the participants’ limited prior exposure to rural school contexts meant that their imagination remained oriented towards a general rather than a rural-specific identity. The community of practice, composed entirely of university-based peers and teacher educators, provided professional but not rural-specific discursive resources. These limitations are not weaknesses of the course itself, but reflections of the structural constraints of pre-service teacher education in China’s Excellent Teacher Program: a single pedagogy course cannot compensate for the lack of direct rural practicum experience, mentorship from experienced rural teachers, or contextualized professional development. Our findings thus suggest that while a well-designed university-based course can initiate identity transformation, it must be complemented by rural-specific practicum experiences to fully develop the knowledge, skills and commitment required for effective rural teaching. Future research should examine how such integrated models can be designed and implemented across different institutional and policy contexts.

## Conclusion

This research demonstrates that a well-structured, holistic English pedagogy course can serve as a productive pathway for TPI reconstruction among rural pre-service English teachers, addressing both their limited pedagogical vision and their affective uncertainties. Through engagement, imagination, and alignment, three mechanisms activated within a discourse-rich community of practice, participants shifted from simplistic and fear-laden conceptions toward professional, confident, and collective professional selves. However, the course’s impact on rural-specific preparedness remained partial, pointing to the need for complementary interventions that directly address the unique conditions of rural teaching.

These findings have practical implications for teacher education programs. We recommend that teacher education programs integrate sustained, discourse-rich pedagogical training with opportunities for authentic rural school experiences. Such experiences, for example, short-term placements or practicum in local rural schools, could help pre-service teachers connect their newly developed professional identities with the realities of rural teaching, thereby reinforcing their confidence to teach in these areas. More broadly, our study offers an international implication: in contexts where extended field placements are not feasible, a campus-based, holistically designed pedagogy course can serve as a practical and effective pathway for rural pre-service teacher identity construction.

Two types of limitations should be distinguished. First, with respect to research design, the research was conducted in a single regional context and with a small sample, which may not fully represent the diverse experiences in other rural areas. Second, concerning the course itself, while the English pedagogy course significantly reshaped participants’ TPI, it alone was insufficient to fully prepare them for the distinctive challenges of rural education, such as professional isolation, limited resources, and multi-grade teaching contexts. This suggests that a well-designed pedagogy course, though effective in identity construction, may need to be complemented by targeted rural placements and contextualized support to translate identity gains into long‑term commitment to rural schools.

Building on these limitations, future research should investigate how teacher education programs can more effectively integrate local rural knowledge and support systems, for example, through mentorship networks, hybrid online-offline communities, or short-term rural practicum experiences, to further strengthen the dedication and professional growth of rural pre-service teachers. Longitudinal studies are also needed to examine whether the TPI growth observed in this course translates into actual retention in rural schools after graduation. Such efforts could yield more effective strategies for teacher retention and enhanced educational outcomes in rural regions.
